# Effects of reduced crude protein diets on footpad dermatitis, necrotic enteritis, and coccidiosis in broiler chickens

**DOI:** 10.1016/j.psj.2026.107442

**Published:** 2026-07-16

**Authors:** Deepak Subedi, Milan Kandel, Jose A. Quinteros, Sonia Y. Liu, Wendy I. Muir

**Affiliations:** a School of Life and Environmental Sciences, Faculty of Science, The University of Sydney, Sydney, NSW 2006, Australia; b Poultry Research Foundation, The University of Sydney, Camden NSW 2570, Australia; c Sydney School of Veterinary Science, Faculty of Science, The University of Sydney, Sydney, NSW 2006, Australia

**Keywords:** Amino acid, Broiler, Reduced crude protein, Footpad dermatitis, Coccidiosis

## Abstract

Reducing dietary crude protein (CP) with balanced amino acid (AA) supplementation has emerged as a practical nutritional strategy to improve the sustainability of broiler production by lowering nitrogen excretion, ammonia emission, and reliance on imported feed ingredients. Beyond these environmental and economic benefits, this review evaluates current evidence on how dietary crude protein levels influence key health and welfare outcomes, including footpad dermatitis (FPD), necrotic enteritis (NE), and coccidiosis in broiler chickens. The available literature indicates that moderate reductions in CP, when essential amino acid requirements are met, consistently reduce nitrogen excretion, litter moisture, litter pH, and ammonia release, thereby improving litter quality and reducing the incidence and severity of FPD and hock burn. While the relationship between reduced CP and NE is inconclusive, moderate CP reduction may alleviate NE-related damage by lowering the flow of undigested protein to the hindgut and reducing substrate availability for *Clostridium perfringens*, its causative agent. But excessive protein reduction and/or inadequate amino acid balance can impair performance and fail to protect gut integrity and increase the risk of NE. Responses are further complicated by concurrent changes in diet composition, particularly increased wheat inclusion and non-starch polysaccharide load, which may elevate digesta viscosity and favour pathogen proliferation when not adequately controlled with enzymes. In coccidiosis, reduced CP diets generally do not worsen outcomes when amino acid supply is correctly balanced, and targeted supplementation with functional amino acids such as arginine, threonine, and glutamine could support gut barrier integrity, immune responses, and recovery. Overall, the benefits of reduced CP feeding depend on the extent of protein reduction, amino acid adequacy, ingredient composition, and disease context. Precisely formulated reduced CP diets can contribute to improved welfare, gut health, and environmental sustainability in broiler production, but further work is needed to define the optimal crude protein and amino acid specifications under commercial disease-challenge conditions.

## Introduction

**Fig. 1:**
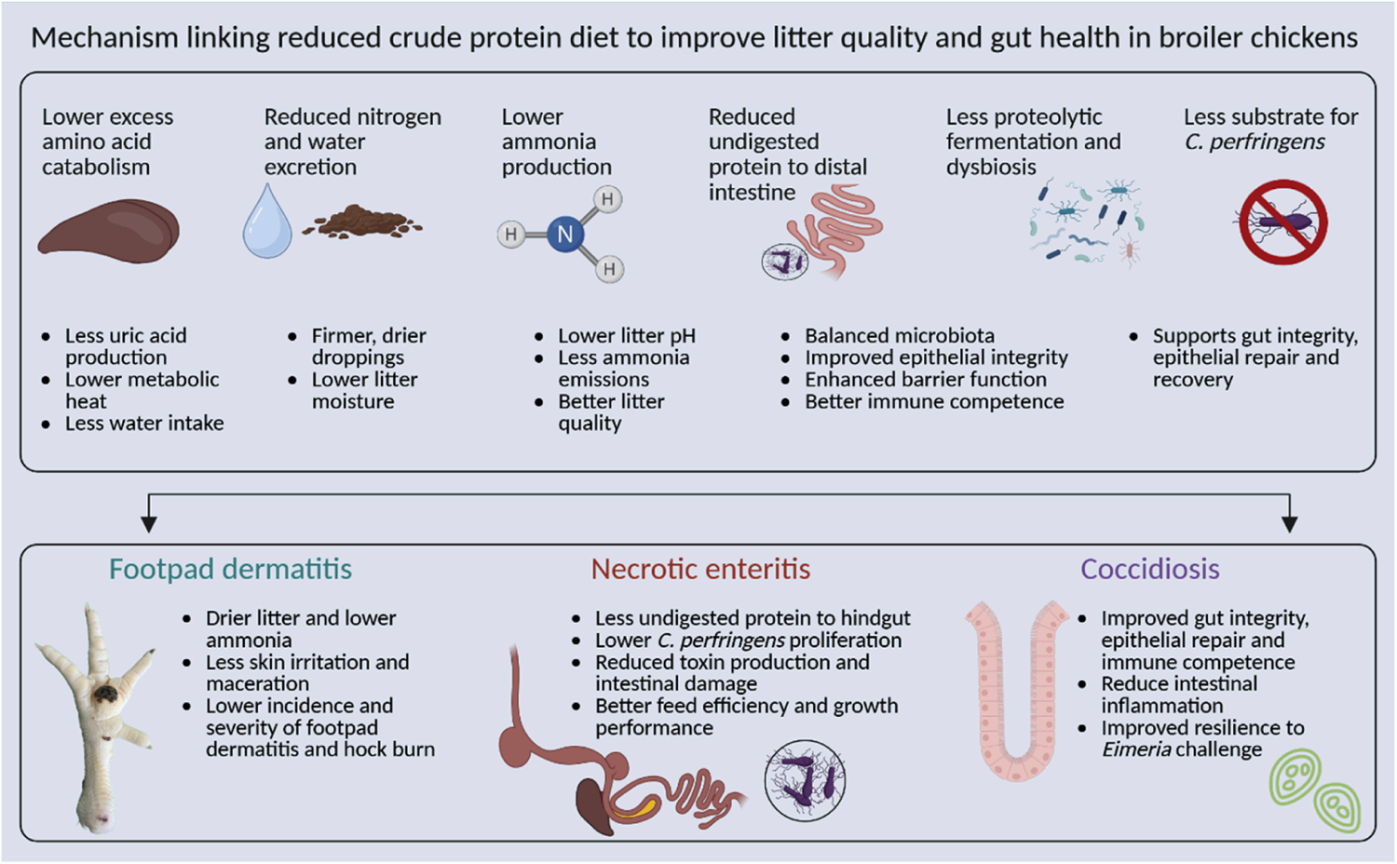
Schematic representation of the mechanisms linking a reduced crude protein diet to improve litter quality, and lower footpad dermatitis, necrotic enteritis and coccidiosis incidence. This Fig. was created with biorender.com (www.biorender.com).

The world’s population is growing by about 0.85% annually and is projected to reach more than 9 billion people by 2050 (Bahar et al., 2020). As global population and incomes continue to rise, poultry meat consumption is expected to increase, with global poultry consumption projected to grow by approximately 21% by 2034 relative to the 2022–24 base period, reaching 173 million tons (Mt) ready-to-cook and accounting for 62% of the additional meat consumed globally (OECD/FAO, 2025). This rapid growth in chicken meat production raises significant concerns regarding sustainability and environmental impact (Bist et al., 2024). These challenges are primarily driven by the limited availability of key protein-rich feed ingredients in some markets, such as soybean, and the contribution of poultry production to anthropogenic greenhouse gas emissions (Wiedemann et al., 2016). Although poultry production emits fewer greenhouse gases than other livestock systems, nitrogen emissions remain a major concern. These include ammonia released into the air, nitrous oxide contributing to global warming, and nitrate leaching into soil and water (Leinonen and Kyriazakis, 2016; Bist et al., 2023). Reducing the crude protein (CP) content supplemented with non-bound amino acids (NBAAs) in broiler diets is a promising approach to enhance the sustainability of chicken meat production and reduce environmental impact (Selle et al., 2020; Liu et al., 2021). Beyond performance and environmental outcomes, reduced CP diets may also affect broiler health and welfare by decreasing nitrogen excretion, litter moisture, ammonia production, and the flow of undigested protein to the hindgut, all of which are relevant to footpad dermatitis (FPD), necrotic enteritis (NE), and coccidiosis (Fig. 1).

According to the broiler nutrition specifications, recommended CP levels for Ross 308 broilers are 23.0% for starter, 21.5% for grower, and 19.5% for finisher phases, and Cobb 500 broilers require 21.0–22.0%, 18.0–20.0%, and 17.0–18.0% CP for the respective phases (Aviagen, 2022a; Cobb Vantress, 2022). Reducing CP by up to 3.0% (30 g/kg) with NBAAs supplementation is generally feasible without major performance loss, but larger reductions often impair feed efficiency and increase fat deposition, measurable via abdominal fat-pad weight (Liu et al., 2021). Although a moderate amount of fat may contribute to meat palatability, excessive abdominal fat is undesirable in commercial broiler production because it represents wasted dietary energy, has low economic value, reduces carcass yield, and could increase feed conversion ratio (FCR) (Fouad and El-Senousey, 2014; Chrystal et al., 2021).

Reducing dietary CP level and optimising the inclusion of free amino acids can lower nitrogen excretion (Nahm, 2007). For instance, an average 35% reduction in nitrogen excretion was found when CP was reduced by 27.3% from 220 to 160 g/kg (Greenhalgh et al., 2020), which could be due to the higher digestibility of NBAAs. This reduction in nitrogen output subsequently limits ammonia formation in the litter, improving litter quality by reducing moisture accumulation and thereby minimising the incidence of FPD and hock burns (Van Harn et al., 2019). FPD is a condition that causes necrotic lesions on the plantar surface of the footpads in growing broilers and turkeys (Shepherd and Fairchild, 2010).

Lowering CP when supplemented with limiting amino acids has also been linked to enhanced intestinal morphology, such as increased duodenal and jejunal villus height and improved villus height: crypt depth ratio, as well as increased ileal volatile fatty acid concentrations (Shazali et al., 2019). It is likely due to reduced undigested protein and lower pathogenic bacterial populations in the hindgut (Apajalahti and Vienola, 2016). In addition, decreased ammonia formation prevents the elevation of the intestinal pH and might decrease the proliferation of *C. perfringens* growth (McDevitt et al., 2006) and consequently, mitigate the impact of NE on broiler performance. NE is a major intestinal disease in poultry that compromises growth, health, and welfare, imposing a substantial economic burden on the global industry, with annual losses estimated at approximately US $6 billion (Abd El-Hack et al., 2021, Li et al., 2017). It occurs when NetB-toxin-producing *C. perfringens* proliferate in response to intestinal disturbances, such as infection with *Eimeria* spp., poor rearing conditions, stress, immunosuppression, and certain dietary components (Moore, 2016; Fathima et al., 2022). These factors damage gut integrity, disrupt microbial balance, and weaken immunity, increasing susceptibility to *C. perfringens* colonisation and NE development.

Similarly, coccidiosis is one of the most prevalent and economically significant diseases in poultry, caused by protozoan parasites of the genus *Eimeria* (Dalloul and Lillehoj, 2006). Depending on the species of *Eimeria* affecting the chicken, clinical signs can vary from intestinal damage and inflammation to increased mortality. Most of them impair production efficiency by reducing feed intake and nutrient utilisation (Teng et al., 2020; Sharma et al., 2022). As seen with coccidiosis, *Eimeria*-induced disruption of gut integrity facilitates the proliferation of *C. perfringens*, predisposing birds to NE and compounding gastrointestinal health challenges. However, feeding a low CP diet to *Eimeria* challenged broilers did not negatively affect intestinal health or growth performance compared to a conventional CP diet (Teng et al., 2021; Liu et al., 2024). Thus, formulating low CP diets for broilers with mild coccidiosis could be an effective strategy for reducing feed costs while maintaining growth performance (Teng et al., 2021).

The previous reviews of reduced CP diets have primarily focused on performance, nutrient utilisation, environmental impact, and sustainability (Aftab et al., 2006; Hilliar and Swick, 2019; Greenhalgh et al., 2020; Liu et al., 2021). However, comparatively less attention has been given to how dietary protein level may influence FPD, NE, and coccidiosis in broiler chickens. Dietary protein concentration may affect these conditions by altering the amount of undigested dietary protein reaching the hindgut, nitrogen excretion, and litter moisture. Undigested protein and amino acids can provide substrates for microbial fermentation, which may increase ammonia production and contribute to unfavourable intestinal and litter conditions (Lemme et al., 2019). Therefore, this review evaluates the current evidence linking dietary protein level with FPD, NE, and coccidiosis in broiler chickens.

## Litter quality and footpad dermatitis (FPD)

Poultry litter consists of bedding mixed with feathers, faeces, and spilled feed or water (Brink et al., 2022) accumulated on the floor of the poultry shed. For optimal bird welfare, the litter must remain dry and friable, as this allows birds to scratch, forage, and dust-bathe (Holt et al., 2023). When litter becomes wet or compacted, the welfare of the broiler declines. Moist litter greatly increases the risk and severity of FPD, with problems typically emerging once moisture exceeds approximately 35% (Sargeant et al., 2019). Litter moisture also drives ammonia production by uric-acid-degrading bacteria, since wet conditions promote microbial activity that converts nitrogenous waste into ammonia, which negatively affects broiler upper respiratory tract integrity and growth performance (Toppel et al., 2019).

Reducing dietary CP lowers ammonia and litter moisture through several interconnected physiological and microbial mechanisms. When birds consume excess protein, the surplus nitrogen is excreted as uric acid, which becomes a major substrate for microbial conversion to ammonia within the litter (Karasawa, 1986). At the same time, low CP diets reduce the amount of undigested protein entering the hindgut, which limits fermentation and osmotic water loss, resulting in firmer, drier droppings (Apajalahti and Vienola, 2016). Conversely, high-protein diets increase metabolic heat and renal excretion, causing birds to drink more, contributing further to wet litter. For example, an increase of 10 g/kg CP has been found to increase water intake by 3% (Francesch and Brufau, 2004). Similarly, an increase in soybean meal raises the litter moisture, which could be attributed to its high potassium level (2.27g/100 g) (Ravindran et al., 2014; Greenhalgh et al., 2020). Lemme et al. (2019) showed that reducing CP from 20.4% to 17.9% increased litter dry matter (from 39.5% to 44.4%) and reduced total N and ammonia-N, demonstrating chemically drier and less irritating litter. Similarly, Son et al. (2024) reported linear reductions in litter moisture (from 65.7% to 60.0%) and pH (from 8.76 to 8.08) as dietary CP decreased, further suppressing ammonia volatilisation and improving bedding friability. In addition, recent meta-analytic evidence provides strong quantitative support for the consistent improvements in litter quality observed under a low CP feeding strategy. Across nine nitrogen mass‑balance studies, broiler litter production averaged 64 g/bird/day, with 62% dry matter, 2.2% nitrogen, and a moderately alkaline pH of 8.5. Reducing dietary CP showed clear linear responses in these parameters: each 1%-point decrease in CP reduced litter mass and litter nitrogen concentration by approximately 3%, increased litter dry matter by 1%-point, and lowered litter pH by 0.13 units (de Rauglaudre et al., 2025).

In poultry production, nitrogen in faeces mainly originates from unabsorbed protein and uric acid, which account for approximately 70% and 30%, respectively (Bist et al., 2023). After excretion, it occurs in several forms, including urea (4-12%), uric acid (40-70%), and residual feed protein (10-40%), with proportions varying among studies and production conditions. These compounds are rapidly hydrolysed into ammonia through microbial enzymatic activity during manure decomposition (Dróżdż et al., 2020). High ammonia concentrations in poultry houses impair bird health and productivity (Olanrewaju et al., 2007). Additionally, it may lead to unsafe or unhealthy working conditions for farmers and farm workers. Ammonia concentrations in poultry houses should be maintained below 25 ppm to prevent adverse health and welfare effects (Bist et al., 2023). Levels above 25 ppm cause ocular and respiratory damage, while chronic exposure leads to organ abnormalities and reduced performance (Quarles and Caveny, 1979). Concentrations greater than 75 ppm reduce broiler growth, meat quality, and muscle gene expression, alongside increased mortality (Miles et al., 2004; Wei et al., 2014).

Reducing dietary CP while supplementing NBAAs has been shown to effectively lower ammonia emissions from poultry housing (Liu et al., 2011; Powers et al., 2006). A 9.9% reduction in weighted crude protein (19.96% versus 17.98%) led to a 21.6% significant decrease in ammonia emissions, dropping from 33.8 mg/kg/day to 26.5 mg/kg/day (Powers et al., 2006). Similarly, on a commercial broiler farm in Queensland, Australia, reducing dietary CP from 21.3% to 19.8% cut ammonia emissions significantly by 27%, dropping from 10.6 g/bird to 7.7 g/bird over a 42-day cycle (Wiedemann et al., 2016). Similarly, reducing dietary CP in broiler breeder diets by 15 g/kg (from around 140 to 125 g/kg) significantly lowered nitrogen excretion by 8%, ammonia emissions by 9%, and total N losses by 11%, without affecting feed or water intake. Overall, each 10 g/kg CP reduction decreased ammonia emissions by about 6% (van Emous et al., 2019). Additionally, in the study by Ospina-Rojas et al. (2012), lowering CP from 22% to 19% significantly reduced litter nitrogen content by approximately 24% and ammonia emission by 37% compared to the control diet. Across meta-analyses, each 1%-point CP reduction decreased N intake by 0.21 g/day (5%), N excretion by 0.20 g/day (11%), and N volatilisation by 0.12 g/day (23%), while lowering the volatilisation rate by 4.2%-points (de Rauglaudre et al., 2025). Similarly, Alfonso-Avila et al. (2022) reported that reducing CP from 19% to 17% cut N excretion by 29% in the grower phase and 18% in the finisher phase, with N efficiency (percentage of dietary nitrogen consumed by the bird that is retained as body protein) improving by 2-3%-points per 1% CP reduction. de Rauglaudre et al. (2023) confirmed these trends, showing 10-14% reductions in N excretion per 1%-point CP decrease when amino acids were controlled. As nitrogen excretion is strongly associated with ammonia formation in poultry houses (r = 0.848; *P* = 0.033), lowering CP in broiler diets can help reduce ammonia emissions (Aderibigbe et al., 2024). This strategy has the potential to enhance bird welfare by mitigating ammonia-related stress and to promote environmental sustainability through reduced nitrogen and ammonia emissions, thereby contributing to improved air, water, and soil quality.

Footpad dermatitis (FPD) or pododermatitis is associated with litter quality, stocking density and overall moisture levels (Alabi et al., 2024). This condition in broilers compromises bird welfare and significantly influences their growth performance, productivity, and behaviour, and it is a common cause of condemnation and downgrading of chicken feet (Nagaraj et al., 2007). The global processed chicken feet market is expected to grow at a compound annual growth rate (CAGR) of 6.8% between 2025 and 2030, underscoring the significant revenue potential of high-quality, unblemished chicken paws (Sims, 2025). Wet litter is the primary risk factor for FPD in broiler chickens (de Jong et al., 2014). Excess moisture softens the skin, making it more susceptible to chemical irritation from ammonia and physical damage, which accelerates lesion development (Berg, 2004). Feeding a low CP diet reduces water intake and uric acid excretion, which helps maintain better litter quality and lowers the incidence of FPD, hock burns, breast skin lesions, and associated skin inflammation (Salahi et al., 2025). For instance, based on the footpad scoring system described by Veldkamp and de Jong (2007), van Harn et al. (2019) found that stepwise reductions in grower and finisher CP improved footpad condition. Flock footpad scores decreased from 143 in the control treatment to 110, 79, and 39 in the CP-1%, CP-2%, and CP-3% treatments, respectively. Similarly, Lambert et al. (2023) reported a marked linear improvement in footpad score at 28 d, declining from 22.3 in the control to 7.1, 5.4, and 0.0 in diets reduced by 1%, 2%, and 3% CP, respectively. Anas et al. (2024) also observed that reducing CP from 20% to 16% significantly decreased footpad scores and ammonia emissions. Furthermore, feeding reduced CP diets to broiler breeder hens significantly improved litter quality and lowered the incidence of FPD (Li et al., 2018).

Likewise, hock burn or hock dermatitis is a form of contact dermatitis that occurs on the skin covering the caudal (rear) aspect of the hock joint (Bessei, 2006). It develops when prolonged contact with wet or soiled litter causes skin discolouration and irritation, which can progress to lesions. During sitting, the broiler’s hocks and breast are in direct contact with the litter. Consequently, excessive litter moisture is a crucial predisposing factor, increasing the likelihood of hock and breast skin dermatitis (Greene et al., 1985). Similar to FPD, feeding a low CP diet has the potential to reduce hock burn in broilers. In a study of Son et al. (2024), the hock burn score decreased from 1.10 at 204.8 g/kg CP to 0.83 at 169.6 g/kg CP, a 24.5% reduction over the range tested. Similarly, broilers fed a low CP diet (158.4 g/kg) reported significantly lower scores for FPD and hock burn damage, compared to those fed a high CP diet (198.0 g/kg) (Anas et al., 2024).

While a few studies have reported minimal or inconsistent effects of reduced CP diet on broiler welfare indicators (Wilhelmsson et al., 2019; Perricone et al., 2024), collective evidence shows that reducing dietary CP while balancing essential AAs significantly decreases nitrogen excretion and ammonia emissions, thereby improving litter conditions and mitigating key welfare issues such as FPD and hock burn.

## Necrotic enteritis (NE)

Necrotic enteritis (NE) is among the most common enteric diseases in poultry and was first documented in a Black Orpington pullet in Australia (Bennetts, 1930). NE is caused by *Clostridium perfringens,* which typically affects chickens between 2 weeks and 6 months of age and is responsible for an estimated global economic burden of approximately 6 billion USD annually (Abd El-Hack et al., 2021, Li et al., 2017). Under normal conditions, *C. perfringens* is present in the intestine at low levels (around 10⁴ CFU/g of digesta). However, when favourable conditions arise, its population can rapidly increase to between 10⁷ and 10⁹ CFU/g of digesta, leading to clinical disease (Drew et al., 2004). Predisposing factors for NE include intestinal epithelial damage, excessive mucus secretion, disruption of gut microbiota, altered gut transit time, and impaired immune status. These changes create a favourable environment for *C. perfringens* proliferation and colonisation, ultimately triggering disease outbreaks (Moore, 2016; Fathima et al., 2022). For decades antibiotic growth promoters (AGPs) were widely used in poultry to control diseases such as NE, but growing concerns over antimicrobial resistance led to strict regulations such as the EU ban (2006) and the U.S. Veterinary Feed Directive (Van Immerseel et al., 2016). Following AGP withdrawal, NE incidence increased significantly, with studies showing much higher clinical and subclinical NE in drug-free flocks compared to conventional systems (Abd El-Hack et al., 2021).

**Fig. 2:**
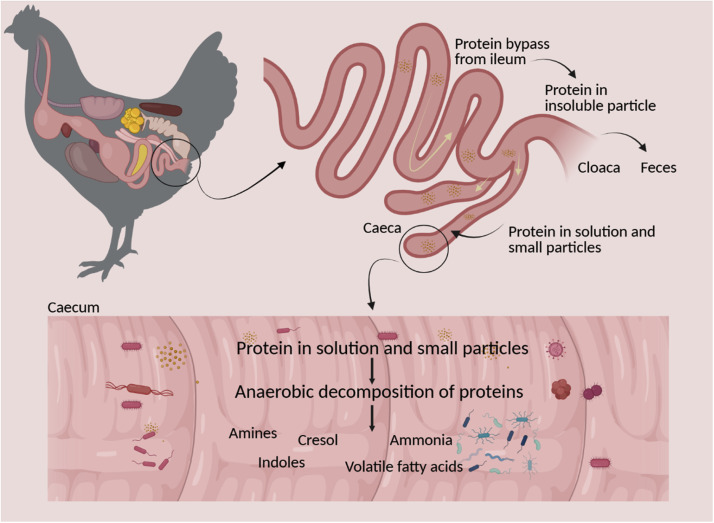
Protein bypass and microbial putrefaction in the caeca of broiler chickens. This Fig. was created with biorender.com (www.biorender.com).

There are two major dietary reasons considered important predisposing factors for the development of NE in broilers. The first is a high CP diet from animal-derived protein sources (Kaldhusdal and Skjerve, 1996; Drew et al., 2004). The second is high dietary inclusion of cereals such as wheat, barley, and rye which are rich in non-starch polysaccharides (NSPs) such as β-glucans or arabinoxylans. These conditions predispose birds to NE by increasing intestinal digesta viscosity and mucus production thereby creating favourable environments for *C. perfringens* proliferation (McDevitt et al., 2006; Shojadoost et al., 2012). In addition, NSPs also elevate water intake, leading to wet litter and promoting bacterial sporulation and litter contamination (Fathima et al., 2022). High dietary CP levels and poorly digestible protein sources increase the flow of protein-bound AAs to the hindgut. These undigested AAs reaching the hindgut can promote the overgrowth of *C. perfringens*, as AA availability influence its proliferation and α-toxin production (Titball et al., 1999). In addition, bypass proteins in the hindgut are fermented by gut microbiota into ammonia, amines, phenols, cresols, and indoles (Fig. 2) (Apajalahti and Vienola, 2016; Yadav and Jha, 2019), which may disrupt intestinal cell turnover and create conditions favourable for *C. perfringens* proliferation, ultimately increasing the risk of NE and negatively affecting broiler growth performance. Nitrogenous degradation products such as ammonia and amines act as weak bases with high pKa values, buffering the gut environment and raising pH. This counteracts the acidification normally caused by fermentation products like acetic and lactic acids, creating conditions that favour *C. perfringens* proliferation (McDevitt et al., 2006). Overall, this may result in compromised intestinal barrier function, diminished nutrient absorption efficiency, and an increased susceptibility to NE.

The level and source of dietary protein also have a strong influence on *C. perfringens* populations in the broiler intestine (Drew et al., 2004). For example, increasing CP from 230 to 400 g/kg raised *C. perfringens* counts significantly, especially in fishmeal diets (cecum: 4.57 to 7.20 log CFU/g) compared to soy protein concentrate (SPC) diets, where it showed smaller increases (cecum: 3.65 to 4.56 log CFU/g). Animal protein sources combined with high protein levels create a more favourable environment for *C. perfringens* proliferation, likely due to elevated glycine and methionine content (Dahiya et al., 2005). Dietary glycine showed a clear quadratic effect on *C. perfringens* growth, with peak cecal counts at approximately 3 to 4% glycine (Dahiya et al., 2005). Birds fed 3.04% glycine had significantly higher *C. perfringens* populations than those on 0.75% or 1.58%, and counts increased by nearly 2 log units when glycine rose from 0.5% to 2.0%. Very high levels of glycine (4%) did not induce further increases in growth of *C. perfringens*, suggesting an optimal range of glycine (between 3.30-3.89%) for proliferation of *C. perfringens* (Dahiya et al., 2005). Additionally, methionine, although not an essential nutrient for *C. perfringens*, can stimulate its growth and sporulation (Muhammed et al., 1975).

Regarding other dietary amino acids, tryptophan has recently shown protective effects against *C. perfringens* challenge. In broilers challenged with *C. perfringens,* supplementation with 0.1% tryptophan improved broiler growth performance, alleviated jejunal injury, enhanced serum immunoglobulins (IgA and IgG), reduced inflammatory responses, improved tight-junction-related gene expression, and upregulated AhR/CYP1A1 signalling. However, 0.2% tryptophan was less effective, suggesting that moderate supplementation with tryptophan is more beneficial than a higher inclusion level under *C. perfringens* challenge conditions (Fan et al., 2025). Arginine also has strong direct evidence in this context, as dietary arginine inhibited intestinal *C. perfringens* colonisation, attenuated mucosal injury, and improved intestinal immune and barrier responses in broiler chickens (Zhang et al., 2017a). Subsequent work further demonstrated that arginine alleviated intestinal inflammatory responses and modulated ileal microbiota in birds challenged with *C. perfringens* (Zhang et al., 2018, 2019). Further, the requirements for threonine increased during subclinical intestinal *Clostridium* infection to support mucin synthesis and intestinal protection under challenge conditions (Star et al., 2012). Glutamine has been shown to alleviate the adverse effects of NE challenge by reducing intestinal lesion scores, improving jejunal morphology, and improving growth performance. Hence, glutamine may act as a functional amino acid during *C. perfringens* associated intestinal stress (Xue et al., 2018), whereby it not only contributes to protein accretion and growth but also to gut integrity, immune competence, antioxidant defence, inflammatory regulation, and resilience under nutritional or disease challenge conditions.

Taken together, the available evidence suggests that amino acids may either exacerbate or alleviate *C. perfringens-*associated enteric stress depending on their functional role. In this context glycine and methionine may be more supportive of bacterial proliferation, while tryptophan, arginine, threonine, and glutamine appear to be more beneficial to the host through enhancement of gut integrity, immune competence, and intestinal repair.

When investigating the impact of different levels of dietary CP on NE, Dao et al. (2022a), predisposed broilers to subclinical NE by administering *Eimeria* on day 9, followed by *C. perfringens* inoculation on day 14 and fed either standard- or reduced CP diets. The reduced CP strategy appeared to improve both gut health indicators and growth performance during the grower phase. The reduced CP diet contained 21.2, 19.4, and 17.4% CP across the starter, grower, and finisher phases respectively, compared with 23.2, 21.4, and 19.4% CP in the standard-protein diet. During the grower phase (d 10 to 24), challenged birds fed reduced CP had higher feed intake (1275 vs 1136 g), greater body weight gain (876 vs 750 g), and lower FCR (1.455 vs 1.514) than challenged birds fed standard CP, with significant challenge × protein interactions for feed intake (*P* < 0.001), body weight gain (BWG) (*P* = 0.002), and FCR (*P* = 0.006). At the intestinal level, feeding reduced CP diets to challenged birds was associated with lower serum FITC-dextran, showing reduced gut permeability, lower caecal *C. perfringens* counts, and greater ileal acetic acid and total short-chain fatty acid concentrations (Dao et al., 2022b).

Interestingly, a much greater reduction in CP Hilliar et al. (2020) did not provide the same benefit as those reported by Dao et al. (2022a). In this study, CP was reduced to 17.1% in the grower phase (7-21 days) and 15.8% in the finisher phase (21-35 days), compared with 21.6 and 19.8% in the standard-protein diets. During 7 to 35d, low protein diets reduced BWG (2228 vs 2434 g; *P* < 0.001) and increased FCR (1.504 vs 1.443; *P* < 0.001), with similar adverse effects observed during the grower phase. Overall, it was concluded that, under the subclinical NE model, reducing dietary CP below approximately 19% during the grower period depressed bird performance. Although increasing amino acid density partly improved the performance outcomes, such as finisher-phase BWG and FCR in challenged birds, it did not fully compensate for the adverse effects of the low protein diets. Thus, these findings demonstrate that reducing CP is not inherently beneficial against NE; the response depends on the level of the CP reduction and the adequacy of AA supply.

In Gharib-Naseri et al. (2021), *C. perfringens* damaged the intestine and reduced bird performance, which was not prevented when feeding a reduced CP diet. Birds fed reduced CP diets received 21.0, 19.5, and 17.5% CP in starter, grower and finisher diets respectively, compared with 23.0, 21.5, and 19.5% CP in the standard diets. NE challenge increased jejunal lesion score (*P* < 0.05), reduced duodenal villus height (*P* < 0.05), and reduced villus height:crypt depth ratio (*P* < 0.01) by day 16, showing clear mucosal injury. Over the full 35-day study period, the reduced CP diets lowered body weight gain (1527 vs 1600 g; *P* = 0.010) and increased FCR (1.552 vs 1.505; *P* = 0.006) relative to standard CP. During the grower phase, reduced CP also compromised FCR (1.426 vs 1.353; *P* < 0.001). Importantly, supplementation with *Bacillus amyloliquefaciens* CECT 5940, a spore-forming probiotic with antimicrobial and enzyme-producing properties, improved body weight gain and FCR and reduced caecal *C. perfringens* load in birds fed reduced CP diets. This response was likely related to improved gut microbial balance and nutrient utilisation, as *B. amyloliquefaciens* supplementation increased beneficial bacterial populations, altered short-chain fatty acid profiles, and improved apparent ileal digestibility of selected amino acids. This study, therefore, suggests that CP reduction alone may be insufficient as a strategy to control NE-associated intestinal damage and performance losses, particularly once intestinal damage has already been established.

Asiamah et al. (2026) identified that the response of broilers to a challenge with *C. perfringens* when fed a reduced CP diet depended on the amino acid balance within the diet rather than the CP concentration alone. Cobb 500 broilers were fed a 19% CP normal protein control diet or 17% CP reduced protein diets with essential-to-total amino acid (E:T) ratios of 0.64 or 0.68 from day 9 to 18. A significant diet × challenge interaction was detected for weight gain (*P* = 0.004) and feed intake (*P* = 0.007), but not for FCR (*P* = 0.231). Under NE challenge, body weight gain did not differ significantly among diets, but, as main effects both reduced CP diets had poorer FCR compared to the control diet (1.361 and 1.375 vs 1.288; *P* < 0.001). At the same time, NE challenge downregulated jejunal TJP1 and GLUT2 (both *P* < 0.001), increased the expression of several nutrient transporter, immune, and apoptotic genes, increased splenic IgA (*P* = 0.029), and altered cecal microbiota structure. Hence, AA optimised reduced CP diets with E:T ratio 0.64 can help maintain growth and partly modulate host and microbial responses during NE but may still fail to fully restore feed efficiency or eliminate intestinal disruption.

The contrasting outcomes among these studies likely reflect differences in both the extent of CP reduction in the diet and the adequacy of amino acid balance. In Hilliar et al. (2020) the more severe reduction in CP may have limited the supply of nonessential amino acids and compromised gut integrity, thereby exacerbating NE challenge. This interpretation is supported by the observation that increasing amino acid density, particularly when both essential and nonessential amino acids were increased, partly improved recovery responses under challenge conditions. In contrast, the more favourable performance observed by Dao et al. (2022a) may be related to a more moderate reduction in CP, together with a lower wheat inclusion compared to the studies of Hilliar et al. (2020) and Gharib-Naseri et al. (2021). Formulating isoenergetic diets with reduced CP inevitably changes the proportions of starch, lipids, and non-bound amino acids. Among these, the greatest shifts may occur in starch and its sources, including corn, wheat, sorghum, and legumes.

In the studies discussed above, wheat inclusion generally increased as CP was reduced, yet phytase and/or xylanase inclusion remained unchanged across treatments, and in Gharib-Naseri et al. (2021), no xylanase was included in any diet. Xylanase supplementation hydrolyses NSPs, particularly arabinoxylans, thereby reducing digesta viscosity, improving nutrient and energy utilisation, and supporting growth performance. Increased wheat inclusion without corresponding adjustment of xylanase supplementation may increase NSP-related effects on digesta viscosity, nutrient availability, and microbial fermentation, thereby influencing the bird performance, intestinal damage, nutrient digestibility, microbial activity, and *C. perfringens* proliferation during NE challenge. For example, in Gharib-Naseri et al. (2021), the finisher standard-protein diet contained 49.2% wheat and 22.9% soybean meal, whereas the reduced CP diet contained 59.1% wheat and 13.5% soybean meal. This reformulation increased arabinoxylan concentration by 19.2%, from 1.82% to 2.17%. Wheat contains substantial amounts of soluble NSPs, particularly arabinoxylans, which increase intestinal digesta viscosity (Friesen et al., 1992; Marquardt et al., 1994). Increased viscosity may slow nutrient diffusion, limit enzyme access to substrates, reduce nutrient digestibility, and favour microbial dysbiosis. Under NE challenge conditions, these changes may increase the availability of undigested substrates for opportunistic bacteria and thereby increase the likelihood of *C. perfringens* proliferation and NE development. In addition, dietary NSPs may promote the growth of *Clostridium* spp. (Moran, 2014), while suppressing beneficial commensals such as *Lactobacillus* spp. (Annett et al., 2002).

It is apparent that further research is required to better understand how cereal grain source, particularly wheat- versus corn-based diet formulation, interacts with dietary CP level and amino acid balance in modulating the development of NE in broiler chickens. Particular attention should also be given to the inclusion and dosage of NSP-degrading enzymes, as reducing CP often necessitates greater wheat inclusion (wheat-based diet), thereby increasing the dietary NSP load, potentially affecting nutrient utilisation and gut health (Kim et al., 2022). Low CP diets do not consistently reduce severity of NE, particularly in the presence of coccidiosis, because shifts in amino acid availability, such as increased glycine concentrations, may support the growth of *C. perfringens*. Although supplemental amino acids may enhance intestinal recovery, field studies are needed to confirm their effectiveness under commercial conditions and in the absence of coccidial challenge (Hilliar et al., 2020).

## Coccidiosis

Coccidiosis is a major enteric disease in broilers caused by protozoan parasites of the genus *Eimeria*. The primary *Eimeria* species responsible for coccidiosis in broiler chickens are *E. acervulina, E. maxima*, and *E. tenella* (Györke et al., 2013). Infection with *Eimeria* damages the intestinal epithelial cells, which leads to inflammation, impaired nutrient absorption, poor growth, and increased susceptibility to NE (Mathis et al., 2024). Coccidiosis exerts a considerable economic impact on the global poultry industry, with annual losses estimated at approximately US $13 billion (Blake et al., 2020). This includes production losses, and the costs associated with prophylaxis and treatment.

As previously mentioned, when formulating reduced CP diets, lowering protein-rich ingredients is often accompanied by higher inclusion rates of wheat, corn, or sorghum. This raises the question as to whether higher wheat inclusion can suppress the effects of an *Eimeria* challenge. It was speculated that feeding whole or coarse wheat may reduce the impact of a coccidial challenge by their stimulation of gizzard development and grinding, which may alter the mechanical disruption, excystation, and passage of *Eimeria* oocysts (Cumming, 1992). While some early studies suggested these possible effects, findings were not consistent (Waldenstedt et al., 2000; Banfield et al., 2002). Interestingly, feeding whole wheat was found to increase lesion severity, oocyst output, mortality, and clinical signs in challenged broilers compared with feeding ground wheat (Gabriel et al., 2003). These variable responses likely reflect differences in bird type, age, *Eimeria* species and dose, wheat form and inclusion level, and assessment criteria (Singh et al., 2015).

In a recent study, moderate dietary wheat inclusion (15%) improved turkey poult growth performance before *Eimeria* challenge but increasing dietary wheat had only a limited influence on the overall progression of coccidiosis after challenge. Although the inclusion of 30% wheat in the diet lowered cecal lesion scores and shifted cecal microbial composition, it did not significantly affect oocyst shedding, and most post-challenge responses including oocyst shedding, gut barrier function, intestinal histology, inflammatory responses, and microbial diversity, were inconsistent or limited (Rafieian-Naeini et al., 2026).

The adverse effects of coccidiosis may be lessened when feeding reduced CP diets that are properly balanced with digestible amino acids, including lysine, methionine, cysteine, threonine, valine, isoleucine, arginine, tryptophan, and glycine-equivalent amino acids. As reducing dietary protein lowers the amount of undigested protein entering the hindgut, this may limit protein fermentation resulting in the production of harmful metabolites such as ammonia, amines, indole, and phenol, which can further impair gut health and immune function in challenged birds (Qaisrani et al., 2015; Apajalahti and Vienola, 2016). Studies have demonstrated that low CP diets do not adversely impact intestinal integrity or growth performance in broilers challenged with *E. maxima, E. tenella, and E. acervulina* (Teng et al., 2021; Liu et al., 2024). Hence, developing reduced CP diets supplemented with crystalline/non-bound amino acids offers a promising strategy to enhance antioxidant capacity and immune defences against coccidiosis, particularly in modern ‘Antibiotic-Free’ and ‘No Antibiotics Ever’ poultry production systems (Teng et al., 2021). Functional amino acids can help alleviate the impact of coccidiosis by restoring nutrient digestibility and supporting immune and oxidative balance. Dietary inclusion of these amino acids can improve nutrient digestibility and maintain growth performance under disease challenge (Chalvon-Demersay et al., 2021) and when fed a reduced CP diet (Teng et al., 2021). Therefore, while reducing dietary CP may decrease the flow of undigested nitrogen into the hindgut and limit harmful protein fermentation, the success of this strategy depends on maintaining an adequate balance of digestible amino acids. Under coccidial challenge, several amino acids have functions beyond protein accretion, including support of mucin synthesis, epithelial repair, antioxidant defence, and immune regulation. Accordingly, amino acids such as arginine, methionine, threonine, glutamine, and branched-chain amino acids warrant specific consideration in reduced CP diets for *Eimeria*-challenged broilers.

### Arginine

Arginine (Arg) is a key functional amino acid which plays a crucial role in protein synthesis and accretion in chickens. Beyond its primary role, Arg also supports secondary physiological functions, including immune modulation, wound healing, and antioxidation activity (Sharma and Kim, 2024). Reducing Arg by 40% in a low CP diet decreased body weight gain by 31% (677 g vs. 991 g) and feed efficiency by 13% (706 vs. 812 g/kg) compared to the low CP control diet (Rochell et al., 2016). However, supplementing Arg at 50% above breeder recommendations (17.5 g/kg calculated digestible arginine) in a reduced CP diet (17.5% CP) was observed to attenuate *Eimeria* induced intestinal permeability, improved immune competence by lowering CD8⁺:CD4⁺ T-cell ratios and enhanced macrophage nitric oxide production, and showed a tendency to upregulate tight junction gene expression, although complete restoration was not achieved (Liu et al., 2023). Under concurrent heat stress and *Eimeria* challenge, Arg supplementation at 50% above breeder recommendations (17.5 g/kg calculated digestible arginine) in a 17.5% CP diet increased villus height, upregulated tight junction proteins including occludin (OCLN) and zonula occludens (ZO1 and ZO2), and elevated immune markers such as macrophage nitric oxide (Liu et al., 2026).

Further, Arg supplementation (7.5 g/kg) in a low CP diet (16% CP) significantly reduced intestinal permeability in *Eimeria* challenged broilers compared with the unsupplemented low dietary CP group (Teng et al., 2021). Arg plays an important role in maintaining gut barrier integrity under enteric or stress-related conditions, likely through its involvement in nitric oxide synthesis and immune modulation (Barekatain et al., 2021). However, Arg did not normalise tight junction gene expression (claudin-1), suggesting its primary benefit may be linked to reducing paracellular leakage rather than directly regulating tight junction proteins (Teng et al., 2021). Supplementing Arg at 50% above the recommended level (17.5 g/kg diet) in a low CP diet (16.5% CP) significantly improved body weight gain and feed efficiency during the acute phase of *Eimeria* infection and enhanced the digestibility of Arg. However, this strategy did not fully restore the expression of intestinal amino acid transporters (Ajao et al., 2024).

### Methionine

Methionine (Met) is an essential and typically the first limiting amino acid in poultry diets. It is vital for growth, protein synthesis and for maintaining intestinal integrity, supporting immune function, and enhancing antioxidant defenses (Liu and Kim, 2023). Met at 50% above breeder recommendations (9.6 g/kg analysed total Met) in a low CP diet (16% CP) during mixed *Eimeria* challenge improved amino acid digestibility and modulated immune-related cytokines (IL-21), but had limited effects on growth performance, intestinal morphology, and oocyst excretion (Taylor et al., 2024). Similarly, supplementing 0.75% (7.5 g/kg feed) Met in a low CP diet (16% CP) exacerbated *Eimeria* infection, as evidenced by increased oocyst shedding, impaired intestinal morphology, and reduced absorption of several amino acids, despite improving Met digestibility (Teng et al., 2021). Under concurrent heat stress and coccidiosis, Met supplementation at 50% above breeder recommendations (9.58 g/kg standardized digestible Met) in a reduced CP diet (17.5% CP) failed to improve growth performance and was associated with increased intestinal permeability (Liu et al., 2026).

Furthermore, in broilers fed a reduced CP diet (17% CP) and challenged with mixed *Eimeria* spp., increasing dietary Met levels from 2.8 to 9.2 g/kg improved post-challenge performance, as reflected by increased BW and BWG and reduced FCR, particularly during the acute infection phase. However, this performance benefit appeared to plateau at approximately 6.0 g/kg Met, whereas total oocyst shedding increased linearly with increasing Met level (Liu et al., 2024). Excess Met downregulates Met/folate metabolism genes, alters one-carbon metabolism that may increase pyrimidine synthesis and potentially favour *Eimeria* replication. Hence, reducing the dietary CP level by 3% with 6.0 g/ kg of Met could maintain the performance and intestinal health of broilers under *Eimeria* challenge (Table 1) (Liu et al., 2024).

**Table 1:** Summary of studies evaluating reduced crude protein diets and non-bound amino acid supplementation in broiler chickens under coccidiosis challenge.

Study	Protein level (normal vs reduced)	*Eimeria* challenge day	*Eimeria* species	Additional amino acids	Growth performance outcome	Gut health / intestinal health outcome
Liu et al. (2026)	Starter (d0-9): common diet; Grower (d9-28): normal 20.0% CP vs reduced 17.5% CP; Finisher (d28–35): common diet	d14	*E. maxima, E. tenella,* and *E. acervulina*.	Arg or Met at 50% above breeder recommendation	Under concurrent heat stress and coccidia challenge, Arg improved BWG, especially during d4–7 post-inoculation, whereas Met was less consistent.	Arg increased OCLN and ZO-1 expression and supported intestinal and immune responses better than Met under combined stress.
Liu et al. (2024)	Starter (d0-14): common diet; Grower (d15-23): normal 20% CP vs reduced 17% CP	d14	*E. maxima, E. tenella,* and *E. acervulina*	Graded Met: 2.8, 4.4, 6.0, 7.6, 9.2 g/kg	Increasing Met linearly improved growth performance at 6 and 9 DPI, but responses were dose dependent.	Higher Met improved some antioxidant and barrier-related responses, but total oocyst shedding also increased linearly with Met; intestinal and metabolic responses were dose dependent.
Ajao et al. (2024)	Starter (d0-9): common diet; Grower (d9-28): normal 18.5% CP vs reduced 16.5% CP; Finisher (d28-35): common diet	d14	*E. maxima, E. tenella,* and *E. acervulina*	Arg or BCAA at 50% above recommended levels	Arg more consistently improved BWG and FCR during the prepatent and acute phases than BCAA.	Supplemental AA increased digestibility of their respective amino acids on d21, but overall gut and tissue responses were variable, with Arg generally more favorable.
Taylor et al. (2024)	Starter (d1-9): common diet; Grower (d9-28): normal 19% CP vs reduced 16% CP; Finisher (d28-35): common diet	d14	*E. acervulina, E. maxima,* and *E. tenella*	Met or Thr at 50% above breeder recommendations	Low CP diets increased feed intake during some phases, but ADG was generally unchanged; Met and Thr had limited overall performance effects.	No significant diet effect on oocyst excretion or intestinal histology; Met and Thr mainly affected immune markers such as IL-10 and IL-21.
Liu et al. (2023)	Starter (d1-9): common diet; Grower (d9-28): normal 20.0% CP vs reduced 17.5% CP	d14	*E. maxima, E. tenella,* and *E. acervulina*	Arg or BCAA at 50% above recommendations	Performance was not the main benefit, but reduced protein alone was not ideal; Arg tended to be more beneficial than BCAA.	Reduced protein increased intestinal permeability, while Arg and BCAA mitigated this; Arg also improved CD8+:CD4+ response and macrophage NO more clearly.
Teng et al. (2021)	Starter (d1-11): common diet; Grower (d12-23): regular 19% CP vs low CP 16% CP	d14	*E. maxima, E. tenella,* and *E. acervulina*	0.75% Arg, Gln, Met, Thr, or all combined	The 16% CP diet did not impair growth versus challenged control.	Arg reduced intestinal permeability, Thr increased duodenal villus height, and Gln/Thr improved tight-junction-related responses; however, Met and the combined treatment aggravated infection severity.
Fernandes et al. (2018)	Pre-challenge/common period: commercial diet d1-21; Experimental period: control commercial diet vs diets with 3% lower CP from d22-42	d22	Challenge via coccidiosis vaccine (20 times) (*E. acervulina, E. maxima, E. praecox, E. tenella, and E. mitis*)	Gln/Glu plus Thr at 0.70, 0.80, or 0.85% digestible Thr	Growth performance was not the major emphasis of the study.	Higher threonine with Gln/Glu improved ileal villus morphology and mucosal preservation, with the best response at 0.85% Thr.
Rochell et al. (2016)	Nine experimental diets: one LCP control diet (modified from Experiment 1) and eight LCP diets with a 40% reduction in Lys, TSAA, Thr, Val, Ile, Arg, Phe + Tyr, or Gly + Ser. 9 experimental diets × 2 *E. acervulina* infection states (uninfected and infected).	d15	*E. acervulina*	A single amino acid or amino acid pair was reduced by 40% relative to the LCP control diet.	The LCP control diet supported the highest growth performance. Reductions in all amino acids except Phe + Tyr impaired growth, with Lys and Val producing the greatest reductions in BWG and feed efficiency.	Dietary AA reductions did not alter infection-induced increases in duodenal IFN-γ, IL-1β, or IL-10 expression.

AA: amino acids; ADG: average daily gain; Arg: arginine; BCAA: branched-chain amino acids; BWG: body weight gain; CP: crude protein; d: day; DPI: days post-inoculation; FCR: feed conversion ratio; Gln: glutamine; Glu: glutamic acid; Gly: glycine; IFN-γ: interferon gamma; IL: interleukin; Ile: isoleucine; LCP: low crude protein; Lys: lysine; Met: methionine; NO: nitric oxide; OCLN: occludin; Phe: phenylalanine; Ser: serine; Thr: threonine; TSAA: total sulfur amino acids; Tyr: tyrosine; Val: valine; ZO-1: zonula occludens-1.

### Threonine

Threonine (Thr) is typically the third limiting amino acid in broiler diets and is essential for protein, mucin, and immunoglobulin synthesis, thereby supporting stress responses, gut epithelial integrity, and intestinal barrier function (Kidd, 2000; Strifler et al., 2024). Reducing Thr by 30 to 40% in low CP diets significantly impairs growth performance and recovery following *Eimeria acervulina* infection, likely due to its critical function in mucin synthesis and epithelial protection (Rochell et al., 2016). In contrast the addition of 0.75% Thr to low CP diets during mixed *Eimeria* challenge improved duodenal villus height, restored tight junction integrity, and enhanced amino acid digestibility, although it did not affect oocyst shedding, indicating structural rather than antiparasitic benefits (Teng et al., 2021). Thr supplementation at 50% above breeder recommendations increased threonine digestibility and upregulated the anti-inflammatory cytokine IL-10 during recovery, but had limited effects on growth performance or lesion scores (Taylor et al., 2024). In broilers fed diets with 3% reduced CP and supplemented with glutamine plus increasing threonine (0.70–0.85%) under *Eimeria* challenge, the higher threonine level (0.85%) preserved ileal villus morphology, reduced crypt depth, and maintained mucosal integrity, whereas the lower threonine level (0.70%) was insufficient to maintain villus-tip integrity in challenged birds (Fernandes et al., 2018).

### Glutamine

Glutamine (Gln) is a conditionally essential and functional amino acid. It has recently received increasing attention due to its dual role as a metabolic substrate and an immune modulator (Liu et al., 2025). It serves as a primary energy source for enterocytes and supports mucosal repair under stress (Wang et al., 2015). Supplementation of 7.5 g/kg Gln in reduced CP (16% CP) diets improved tight junction integrity by reversing claudin-1 upregulation and enhanced amino acid digestibility during mixed *Eimeria* infection, thereby supporting epithelial recovery but without reducing oocyst shedding (Taylor et al., 2024). Similarly, inclusion of Gln and glutamic acid in diets with 3% reduced CP, together with higher threonine levels (7.07 to 8.48 g/kg), preserved ileal villus morphology, reduced crypt depth, and maintained mucosal integrity in broilers challenged with *Eimeria* spp., with the greatest response observed at 8.48 g/kg digestible threonine (Fernandes et al., 2018). These findings suggest that glutamine/glutamic acid may support enterocyte metabolism and epithelial repair under enteric stress, particularly when adequate digestible threonine is available for mucin synthesis and intestinal barrier maintenance (Kim and Kim, 2017).

### Branched-chain amino acids

Branched-chain amino acids (BCAA), including valine, isoleucine, and leucine, are important for protein synthesis, immune cell metabolism, energy production, cellular signalling, and gut health (Zhang et al., 2017b). Leucine activates protein synthesis through the mTOR pathway, whereas valine and isoleucine are often considered the fourth and fifth limiting amino acids in corn-soybean meal-based broiler diets (Kidd et al., 2021; Ajao et al., 2024). Valine deficiency was among the most growth-limiting amino acid reductions tested in a 16.7% CP diet, alongside reductions in Lys, TSAA, Thr, Ile, Arg, Phe + Tyr, and Gly + Ser, highlighting the importance of maintaining adequate standardized ileal digestible Val in reduced CP diets (Rochell et al., 2016). In a 17.5% CP diet, BCAA supplementation, providing standardized digestible Leu, Ile, and Val at 20.1, 11.4, and 13.2 g/kg, respectively, partially mitigated *Eimeria*-induced intestinal permeability and enhanced macrophage nitric oxide production, although these responses were less pronounced than those observed with Arg supplementation (Liu et al., 2023). Overall, BCAA may contribute to immune regulation and intestinal integrity during *Eimeria* challenge, but current evidence suggests that their protective effects are more modest than those of Arg.

## Conclusion

Reduced CP diets have considerable potential to improve litter quality, lower nitrogen excretion and ammonia emission, and alleviate FPD in broiler chickens. However, their effectiveness in reducing the impact of NE and coccidiosis appear to depend on the magnitude of CP reduction, adequacy of digestible amino acids, and overall diet composition. The central strategy is therefore to formulate reduced CP diets that maintain appropriate digestible amino acid balance while reducing undigested protein flow into the hindgut, thereby limiting nitrogenous substrates for harmful microbial fermentation and supporting intestinal integrity during enteric challenge. Successful implementation will require the development of ideal amino acid ratios specifically suited to reduced CP diets, together with a better understanding of the roles of functional and nonessential amino acids under enteric challenge. These responses should also be interpreted within the broader context of starch-protein digestive dynamics, the relative contributions of protein-bound and non-bound amino acids, cereal inclusion, dietary NSP content, and enzyme supplementation. Overall, properly formulated reduced CP diets remain a promising nutritional approach to improve broiler gut health, welfare, and sustainability in chicken-meat production.

## Author contribution

Conceptualization: DS, MK, SYL, and WIM; writing – original draft: DS; writing – review and editing: DS, MK, JAQ, SYL, and WIM; Visualization: DS; supervision: JAQ, SYL, and WIM. All authors have read and approved the final version of the manuscript for publication.

## Disclosures

The authors declare no conflicts of interest.
